# Different Lower Extremity Arterial Calcification Patterns in Patients with Chronic Limb-Threatening Ischemia Compared with Asymptomatic Controls

**DOI:** 10.3390/jpm11060493

**Published:** 2021-05-31

**Authors:** Louise C. D. Konijn, Richard A. P. Takx, Willem P. Th. M. Mali, Hugo T. C. Veger, Hendrik van Overhagen

**Affiliations:** 1 Department of Diagnostic and Interventional Radiology, Haga Teaching Hospital, Els Borst-Eilersplein 275, 2545 AA The Hague, The Netherlands; h.voverhagen@hagaziekenhuis.nl; 2 Department of Diagnostic and Interventional Radiology, University Medical Center Utrecht and Utrecht University, Heidelberglaan 100, 3584 CX Utrecht, The Netherlands; r.a.p.takx@umcutrecht.nl (R.A.P.T.); w.mali@umcutrecht.nl (W.P.T.M.M.); 3 Department of Vascular Surgery, Haga Teaching Hospital, 2545 AA The Hague, The Netherlands; h.veger@hagaziekenhuis.nl

**Keywords:** chronic limb-threatening ischemia, peripheral arterial disease, calcification pattern

## Abstract

Objectives: The most severe type of peripheral arterial disease (PAD) is critical limb-threatening ischemia (CLI). In CLI, calcification of the vessel wall plays an important role in symptoms, amputation rate, and mortality. However, calcified arteries are also found in asymptomatic persons (non-PAD patients). We investigated whether the calcification pattern in CLI patients and non- PAD patients are different and could possibly explain the symptoms in CLI patients. Materials and Methods: 130 CLI and 204 non-PAD patients underwent a CT of the lower extremities. This resulted in 118 CLI patients (mean age 72 ± 12, 70.3% male) that were age-matched with 118 non-PAD patients (mean age 71 ± 11, 51.7% male). The characteristics severity, annularity, thickness, and continuity were assessed in the femoral and crural arteries and analyzed by binary multiple logistic regression. Results: Nearly all CLI patients have calcifications and these are equally frequent in the femoropopliteal (98.3%) and crural arteries (97.5%), while the non-PAD patients had in just 67% any calcifications with more calcifications in the femoropopliteal (70.3%) than in the crural arteries (55.9%, *p* < 0.005). The crural arteries of CLI patients had significantly more complete annular calcifications (OR 2.92, *p* = 0.001), while in non-PAD patients dot-like calcifications dominated. In CLI patients, the femoropopliteal arteries had more severe, irregular/patchy, and thick calcifications (OR 2.40, 3.27, 1.81, *p* ≤ 0.05, respectively) while in non-PAD patients, thin continuous calcifications prevailed. Conclusions: Compared with non-PAD patients, arteries of the lower extremities of CLI patients are more frequently and extensively calcified. Annular calcifications were found in the crural arteries of CLI patients while dot-like calcifications were mostly present in non-PAD patients. These different patterns of calcifications in CLI point at different etiology and can have prognostic and eventually therapeutic consequences.

## 1. Introduction

Recent studies have shown that calcification across the vascular wall of patients with peripheral arterial disease (PAD) and chronic limb-threatening ischemia (CLI) play an important role [1,2,3]. These calcifications are of clinical importance since they are associated with symptoms, treatment outcome, and mortality [4,5,6]. These studies have also shown that in PAD and CLI, two different types of calcifications can be found: (1) calcifications based on calcified atherosclerotic plaques located in the intimal layer of the vessel wall and (2) calcification of the tunica media and internal elastic lamina of the vessel wall as a separate metabolic disease, causing stiffness and limit remodeling [7].

Medial calcifications are not only present in the arteries of the lower extremities but also for example in the carotid siphon and in the arteries of the breast [8,9,10]. However, medial calcifications are almost absent in the coronary arteries. Although the differentiation between intimal and medial calcification on a CT scan is not completely reliable, annular calcifications most likely represent medial calcifications.

Medial wall calcification is an active metabolic process with bone progression proteins, instead of solely deposition of bisphosphonates in the arterial walls [7,11]. Medial calcifications can be reversible over time as showed in the arteries of the breast, for example, after kidney transplantation, but not all patients have a clear cause for regression of these calcifications [8,12]. Thus, regression of calcifications can presumably occur partially. Indeed, a histological study of the arteries of the lower limbs found osteoclasts, however scarce [13]. Extensive regression of calcifications therefore does not seem plausible.

It has been shown that medial calcifications can be halted by bisphosphonate etidronate in patients with pseudoxanthoma elasticum [14,15], a rare monogenetic disease resulting in medial calcifications of the arteries. Since we know that CLI patients with complete annular calcifications, most likely medial arterial calcifications, have worse survival than patients without these calcifications, treatment of these patients with etidronate could therefore be a therapeutic option.

Since it is known that vascular calcifications also occur in asymptomatic people [16], we wanted to compare CLI patients with these asymptomatic patients and investigate whether the calcification pattern perhaps could be associated with the symptomatology in CLI patients.

Therefore, the primary aim of this present study was to examine differences in presence, severity, and characteristics of arterial calcifications in the lower extremities between CLI patients and patients without known PAD using CT.

## 2. Materials and Methods

### 2.1. CLI Patients

This study consisted of data from two trials with CLI patients. The PADI trial (Percutaneous transluminal Angioplasty and Drug-eluting stents for infrapopliteal lesions in critical limb ischemia), and the PADI Imaging Trial.

From the PADI trial extensive study details and mid- to long-term results have been published elsewhere [17,18,19,20]. Briefly, the PADI Trial was an investigator-initiated prospective, multicenter RCT in CLI patients due to infrapopliteal pathology to assess the value of drug eluting stents (DES) compared to the current reference treatment with bare-metal stents (BMS). Included in the PADI trial were 137 patients. DES provided better 6-month patency rates and less amputations after 6 and 12 months. From these 137 patients 87 patients underwent a CT angiography of the lower extremity and were included in the present study. Patients were scanned on a 256-slice CT scanner (Siemens Definition Flash Scanner, Siemens Healthineers, Forchheim, Germany). Slice thickness was set between 0.625 and 1 mm.

The subsequent PADI Imaging Trial was an investigator-initiated prospective study developed to investigate atherosclerosis and arteriosclerosis in the whole body in patients with CLI. Patients with CLI were recruited in the outpatient clinic of the department of vascular surgery in a large teaching hospital in The Hague, the Netherlands. Patients were excluded from the study if they were unable to give consent, if they were younger than 18 years, if patients were allergic for intravenous contrast. All included patients gave written consent. Extensive clinical assessment, routine cardiovascular laboratory tests, and a whole-body spectral CT were done including a CT angiography of the lower extremities, which was used for this current study. Patients were scanned on a 256-slice CT scanner (Siemens Definition Flash Scanner, Siemens Healthineers, Forchheim, Germany). Slice thickness was set between 0.625 and 1 mm. All 43 participants in the PADI Imaging Trial enrolled to date were included in the current study.

A total of 130 CLI patients from the PADI Trial and PADI Imaging Trial were analyzed in this study.

### 2.2. Non-PAD Patients

As control group we used patients from a different study without known vascular disease as stated in their electronic patient file. Patient details have been published elsewhere [21]. In short, patients with an indication for a full-body PET-CT because of melanoma, infection, unknown fever, or lymphadenopathy were selected. These scans were matched to medical record data for cardiovascular risk factors and symptomatic PAD status. A total of 204 patients without known PAD were included in this study.

### 2.3. Study Approval

The medical ethical board of the participating centers approved the prospective PADI Imaging Trial (Unique identifier number: NL64059.098.17) and to re-use data from the PADI trial (ClinicalTrials.gov trial register Unique identifier number: NCT00471289) as a post hoc analysis.

Regarding the patients without PAD, the medical ethical board of the University Medical Center Utrecht (UMCU) waived review because of its non-invasive, retrospective character (study number: 17-897/C). This study was also approved by the ethical board of the Haga Teaching Hospital (study number: T18-003).

### 2.4. Baseline Measurements and Definitions

The following variables were included for analysis: age, gender, diabetes mellitus (DM), weight, current smoking status, systolic blood pressure, diastolic blood pressure, and renal function (eGFR). Obesity was defined as a body mass index (BMI) ≥ 30 kg/m^2^. Hypertension was defined as a systolic blood pressure at admission >140 mmHg or diastolic blood pressure of >90 mmHg. Chronic kidney disease (CKD) was defined as an eGFR < 60 mL/min/1.73 m^2^, mildly decreased kidney function to renal failure (G3a-G5) [22]. We also showed severely decreased kidney function (G4-G5) with a cut-off value of <30 mL/min/1.73 m^2^, since this is the best prognostic factor in patients with CLI [23].

### 2.5. Calcification Assessment

**Figure 1:**
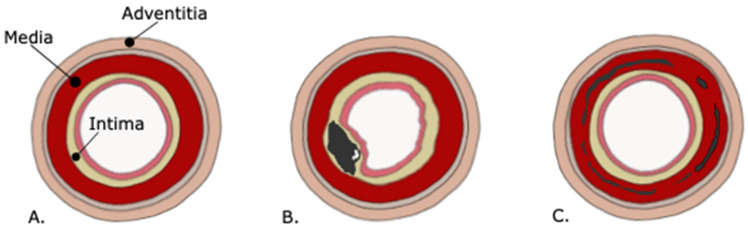
(**A**) Wall layers in a normal artery from inside to outside: endothelium, tunica intima (internal elastic membrane and fibrocollagenous tissue), tunica media (smooth muscle), tunica adventitia (external elastic lamina and fibrocollagenous tissue). (**B**) Calcifications in the intimal wall: thick and non-annular. (**C**) calcifications in the medial wall and in the internal elastic lamina: thin and annular.

**Figure 2:**
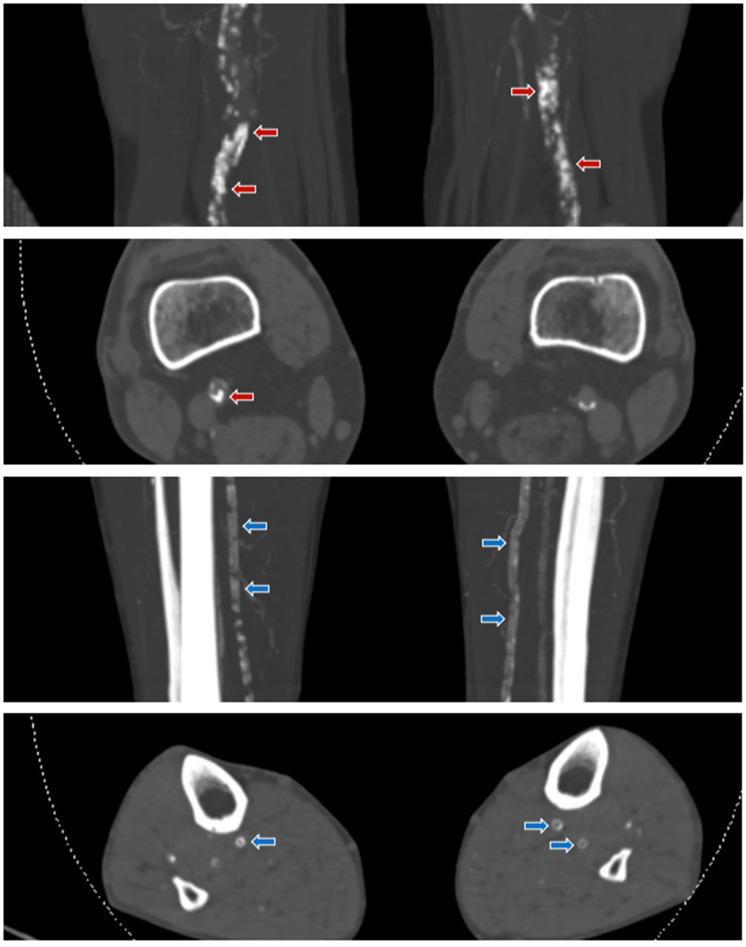
Examples of different calcification patterns in patients with CLI. Shown are coronal MIP and axial 3 mm CT angiography images of the lower extremities. A. Top two figures: the red arrows in femoropopliteal arteries showing irregular/patchy, thick, and non-annular calcifications corresponding to a dominant intimal calcification pattern. B. Bottom two figures: the blue arrows in the crural arteries showing continuous, thin, and annular calcifications corresponding to a dominant medial calcification pattern.

All patients underwent a CT scan of the lower extremities and were evaluated on 3 mm slice thickness reconstructions. For evaluation of arterial calcifications, bone settings were used for all CT exams (Window Settings: Window = 300 Hounsfield Units; Width = 1600 Hounsfield Units). This made it possible to distinguish between calcium and other densities on both CT angiographies and non-contrast CT. Both the femoropopliteal and crural arteries were scored. Vascular calcification patterns were examined in a semi-quantitative way; severity (absent, mild, moderate, severe), annularity (absent, dot(s), <90°, 90–270°, 270–360°), thickness (absent, ≥1.5 mm, <1.5 mm), and continuity (indistinguishable, irregular/patchy, or continuous). In case more patterns were present, the most dominant characteristic was chosen to score. Figure 1 and Figure 2 provide different types of calcifications and illustrative examples of the CT scoring system. Extensive details on the calcification measurements are described elsewhere [21] and is based on the recently developed and CT-histologically validated score for the carotid siphon by Kockelkoren et al. with a proven reproducible scoring system (inter-observer kappa 0.54–0.99) [24,25]. All measurements were performed by a radiology resident with more than 4 years of experience with the scoring system (LCDK) who was blinded to the patients’ clinical data. In one of our other studies with a similar set of CLI patients with Fontaine 3 and 4, test–retest reliability was calculated based on second reader measurements scored by a senior radiologist (WPThM) with over 40 years of radiological experience. Cohen’s weighted Kappa tests (Κw) showed good agreement of interreader test–retest reproducibility. Κw values were for severity 0.72 (95% CI 0.55–0.90, *p* < 0.001), annularity 0.77 (95% CI 00.58–0.95, *p* < 0.001), thickness 0.65 (95% CI 0.29–1.01, *p* < 0.001), and continuity 0.62 (95% CI 0.31–0.94, *p* < 0.001).

### 2.6. Statistical Considerations

#### 2.6.1. Case Matching

Due to an age difference of approximately 9 years in the CLI (cases) and non-PAD patients (controls), age matching was performed. The intention was to construct patient groups with a comparable age, as the occurrence of arterial calcification is age-dependent. The match tolerance (fuzz) factor was set on 5 years. There were 118 matches, 98 patients were excluded of further analysis. This resulted in a final sample of 236 patients, whereof 118 patients with CLI and 118 patients without clinical known PAD. Matching was performed blinded to outcomes.

#### 2.6.2. Statistical Analysis

Descriptive data are presented as mean ± standard deviation (SD) for normally distributed continuous variables, for non-normally distributed continuous variables as median (interquartile range (IQR)). Regarding categorical variables, characteristics were presented as number (percentages). Groups were compared using the Chi-squared test for categorical variables and the Student’s t test for continuous variables.

The prevalence of different calcifications characteristics (severity, annularity, thickness, and continuity) in the femoropopliteal and crural arteries was plotted graphically for both absolute numbers and percentages.

Regarding evaluation of similarities and differences between these CT calcification characteristics for both the femoropopliteal and crural arteries, patients without calcifications were excluded from this analysis (4 CLI patients and 57 non-PAD patients). Of the remaining 114 CLI patients and 61 non-PAD patients with any calcifications, type, and pattern analysis were performed.

Binary multiple logistic regression was performed to evaluate these different CT calcification characteristics. To adjust for confounding, correction for gender was performed. A *p*-value of less than 0.05 was considered to be significant. Data analysis was carried out using SPSS version 27.0 (IBM Corporation, New York, NY, USA).

## 3. Results

### 3.1. Baseline Characteristics

Mean age was 74 years (range 40–95; SD 11). There were 61% (n = 236) male patients. Patients with CLI were significantly more likely to be male, had a higher prevalence of diabetes mellitus, smokers, hypertension and lower eGFR. Baseline characteristics and comorbidities of both CLI and non-PAD are shown in Table 1.

### 3.2. Prevalence of Lower Extremity Arterial Calcifications

The prevalence of any calcification in the crural arteries was 97.5% (115/118) in CLI patients and 55.9% (66/118) in non-PAD patients, *p* < 0.005. In the femoropopliteal arteries, the prevalence of calcifications was 98.3% (116/118) in CLI patients and in non-PAD patients 70.3% (83/118), *p* < 0.005.

### 3.3. Differences in Lower Extremity Arterial Calcification Patterns between CLI and Non-PAD Patients

Patients without calcifications were excluded for further analysis. This resulted in the CLI patient group in the loss of only three (2.5%) patients without crural arterial calcifications and only two patients (1.7%) without femoropopliteal arterial calcifications. In the non-PAD group, 52 (44.1%) patients without crural arterial calcifications were excluded from analysis and 35 (29.7%) non-PAD patients without femoropopliteal arterial calcifications. Baseline characteristics of these subpopulations were not markedly different on any of the variables compared to the primary case-match cohort of 236 patients. These baseline characteristics are shown in Table S1. A further subdivision in baseline characteristics between patients with calcifications and without calcifications in non-PAD patients, showed that patients with calcifications in this group were older, more likely to be male, smoker, and had more often hypertension. See Tables S2 and S3.

**Figure 3:**
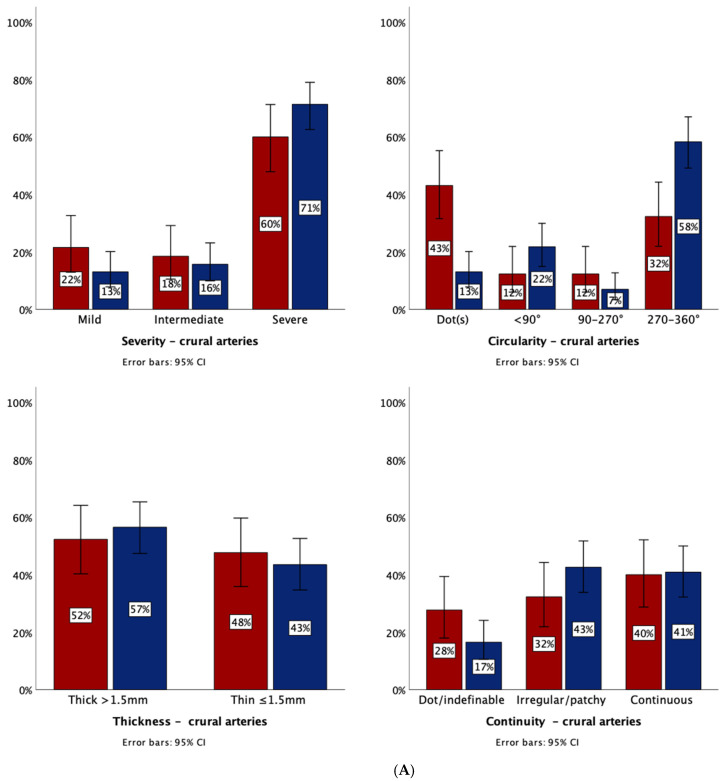

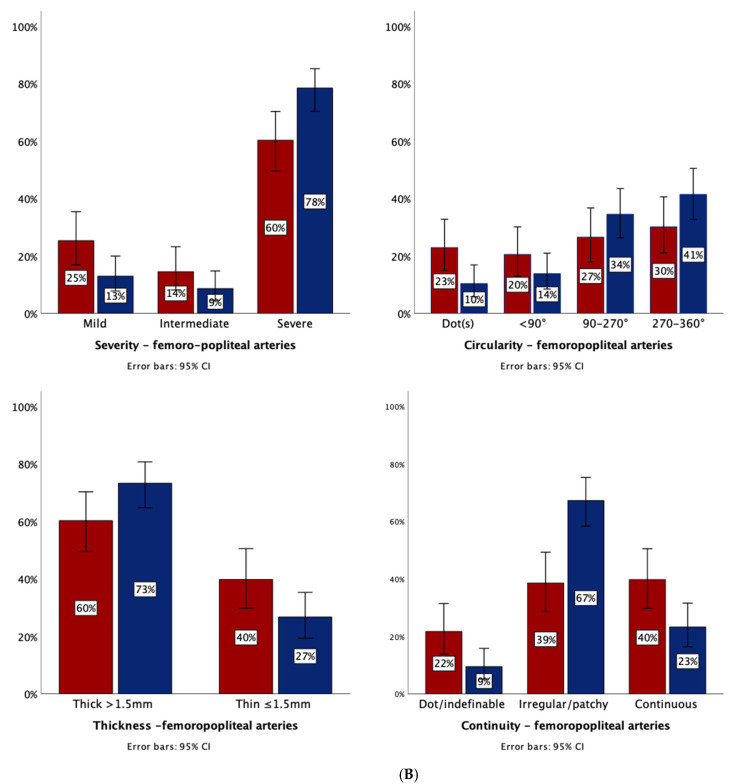
(**A**) CT calcification characteristics in the crural arteries as percentage of total number of age-matched patients. Red: non-PAD patients (n = 65). Blue: CLI patients (n = 115). (**B**) Severity, annularity, thickness, and continuity in the femoropopliteal arteries as percentage of total number of patients. Red: non-PAD patients (n = 83), blue: CLI patients (n = 116).

Comparing all patients with lower extremity arterial calcifications in the crural arteries, most calcifications were severe (CLI vs. non-PAD: 71.3% (82/115) vs. 60.0% (39/65) without a significant difference between these two patient groups. The majority of femoropopliteal arteries were also severely calcified with 78.4% (91/116) in CLI patients compared to 60.2% (50/83) in non-PAD patients, here, a significant OR was found (OR 2.40, 95% CI 1.29–4.48, *p* = 0.006). See Figure 3A,B for stacked graphs and Table 2 and Table 3 for corresponding logistic regression analysis. See Tables S4 and S5 for the severity of calcifications by decade.

**Figure 4:**
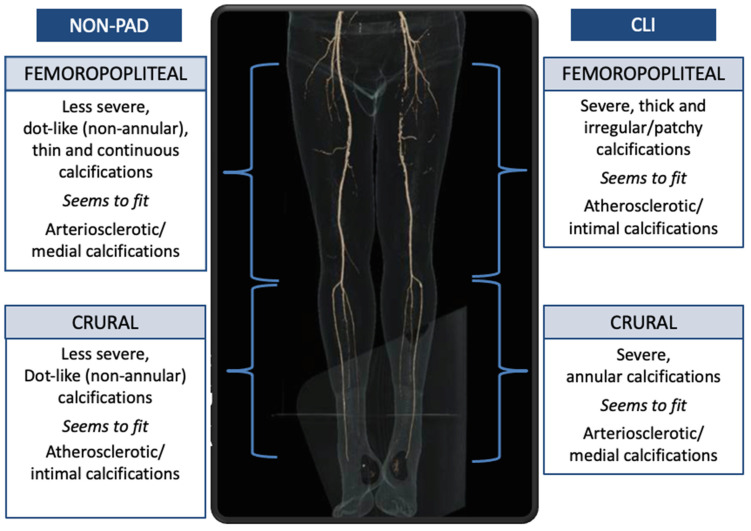
Schematic representation of the different calcification patterns between CLI and non-PAD patients.

Next, the different calcification characteristics of annularity, thickness and continuity were compared between CLI and non-PAD patients and its results are schematically shown in Figure 4.

In the crural arteries, CLI patients had significantly more complete annular calcifications (58.3% (67/115)) than in non-PAD patients (32.3%, 21/65, OR 2.92, 95% CI 1.55–5.44, *p* = 0.001). Dotted (non-annular) calcifications are more frequently found in non-PAD patients, with an OR of 0.20. No significant OR were found for all other calcification characteristics with respect to the crural arteries.

In the femoropopliteal arteries, CLI patients had irregular/patchy calcifications in 67.8% (78/116), significantly more than non-PAD patients with 38.6% (32/83), OR 3.27, 95% CI 1.82–5.89, *p* < 0.005). Moreover, CLI patients had significantly more thick calcifications in the femoropopliteal artery with 73.3% (85/116), compared to non-PAD patients with 60.2% (50/83, OR 1.81, 95% CI 1.09–3.30, *p* = 0.05). All other calcification characteristics the femoropopliteal arteries did not yield significant differences.

### 3.4. Sub Analyses CKD and DM

Since DM and CKD are present in a high percentage of CLI patients and have a substantially worse prognosis than patients without these characteristics, sub-analyses were performed to test the specific subcategories DM and CKD. However, the patient numbers of these sub-analyses were low (see baseline Table 1 for an overview of these numbers), so, *p*-values could not be calculated.

There were no substantial differences between patients with and without DM in non-PAD patients and those with CLI. In the CLI group with CKD, annularity was more frequent (65.4% vs. 54.3%) but the difference with non-CKD CLI patients was small.

## 4. Discussion

In this matched case-control study, the features of arterial calcifications in the arteries of the lower extremities between patients with CLI and non-PAD patients without known vascular disease were investigated using CT imaging.

The principal finding of this study is that in the crural arteries CLI patients have predominantly annular calcifications, while in non-PAD patients these calcifications are mainly dot-like. Second, almost all patients with CLI have calcifications in the femoropopliteal and crural arteries, while the controls without known vascular disease had any calcifications in less than two-thirds of the cases with a considerable difference between the femoropopliteal and crural arteries.

Annular calcifications are mainly found in the crural arteries of CLI patients have been more frequently related to medial arterial calcifications, while dot-like calcifications are mainly found in the crural arteries of non-PAD patients and seem related to atherosclerotic intimal calcifications. There are however no histologic-CT imaging correlation studies available for the crural arteries. However, a histologic-CT imaging study done in the intracranial internal carotid artery (carotid syphon) confirmed that annular calcifications are indeed more likely located in the medial layer of the arterial wall, while the dot-like ones were related to calcifications in atherosclerotic plaques [10].

Our findings are consistent with several histopathological studies showing that in the crural arteries, medial arterial calcifications play a prominent role in CLI patients. Soor et al. showed that the crural arteries in the majority of CLI patients contain medial calcifications (51–100% annularity) and do not have severe atherosclerosis [3]. O’Neill et al. found medial calcifications in 72% of the arteries of the amputation specimen, while atheromas were present in only 23% [26]. More recently, a histopathological post-mortem study analyzing especially CLI patients showed that medial calcifications occur significantly more frequently in the crural than in femoropopliteal arteries (OR 2.89, *p* = 0.08) [2]. These findings are quite different from the findings in the coronary arteries where medial calcifications are rarely found. In our cohort, only three patients had no crural vessel calcifications (2.5%) and only 2 patients (1.7%) had no femoropopliteal arterial calcifications. An occlusive thrombus and/or partially atheromatous wall changes may have played a role in these few patients with CLI.

Previous studies have shown that patients with medial calcifications have a poor prognosis [27,28,29]. Recently, we also showed that CLI patients with complete annular calcifications in the lower extremity had a significantly worse all-cause mortality compared to the group without complete annular calcifications [30]. Another study has shown that patients with a higher degree of annular calcification in the abdominal aorta is associated with a higher all-cause mortality (29).

It was recently suggested that medial calcification and atherosclerotic intimal disease can both be present in the arteries of the lower extremities in PAD patients, and that medial calcification prevents the arteries of the lower extremities from remodeling causing obstructive disease in PAD [31]. In our study, we were unable to demonstrate the presence of the two types due to the fact that when describing the characteristics, we always noted only the most common type. As a result, there was no room for less common calcifications. It may be that atherosclerotic disease could lead to much less severe obstructive disease only in the crural arteries, and the presence of both atherosclerotic and medial disease could lead to much more severe PAD.

In the femoropopliteal arteries of CLI patients we found remarkably severe, irregular/patchy, and thick calcifications, while in non-PAD patients, mild, thin, dot-like, and continuous calcifications are found. So, these findings are different from those in the crural arteries where in CLI patients annular medial type of calcifications were found and in non-PAD patients a dot-like atherosclerotic type. Calcification pattern differs in the different vascular territories.

We did not find a difference in calcification pattern between diabetic and non-diabetic patients in CLI and non-PAD patient groups. Such a difference was found in CKD patients but the difference was small and could not explain the high percentage of annular lesions. However, we should be careful with the interpretation since the numbers in this sub analysis were small.

Intimal calcifications are mainly related to atherosclerotic disease and medial calcifications are a metabolic disease due to an imbalance between pro- and anti-calcification agents. These two diseases therefore justify different therapeutic approaches. Intimal calcifications warrant more classical anti-atherosclerotic medication while medial calcification could be halted by calcification inhibitors such as etidronate. The recent TEMP trial was conducted in a group of PXE patients with severe medial calcified arteries [14,15]. After one year of treatment with etidronate, progressive calcification in the femoral arteries was stopped compared to controls, without significant side effects such as osteonecrosis being found. Therefore, we think that a similar study could be considered to perform in this severely affected patient group of CLI patients.

## 5. Strengths and Limitations

One of the major strengths is that this study compared arterial calcifications in patients with and without symptomatic peripheral vascular disease using CT. The use of CT makes prediction and longitudinal studies of PAD severity through calcification characteristics easier in the long term. A second important strength is that age has been corrected by means of case-matching. Age is a known important confounder of CLI research, and case-matching reduced the possibility of confounding.

However, the study also has its limitations. First, it remains difficult to measure an entire artery with an arbitrary three-, four-, or five-point score. This is certainly the case for CLI patients with a fair number of calcifications in the specific lower extremity arterial territory and therefore both (medial and intimal) patterns are often present. In this case, the most dominant characteristic was chosen to score.

Second, it is remarkable that of the patients without known PAD, there are relatively many patients with severe calcifications. This means that we may have chosen a too low cut-off point for severity of calcifications. Third, the CLI group underwent contrast enhanced CT and the non-PAD group unenhanced CT and therefore we might have underestimated the calcification burden in the CLI group and missed some thin annular calcifications because these may be less recognizable on CT with contrast than CT without contrast. If so, the reported association and findings could be stronger.

Finally, we cannot be completely sure that the non-PAD patients did not have any atherosclerotic disease since no angiograms were made and therefore the lumen was not examined. However, no clinical symptoms were present in these patients and therefore these patients were considered non-PAD patients.

## 6. Conclusions

This study shows that in the arteries of the lower extremities in CLI patients, any arterial calcification is almost always present. In non-PAD patients however, only two third of patients have any calcifications. Most calcifications are severe. In the crural arteries CLI patients have an annular type of calcifications as seen in medial calcifications, while non-PAD patients have a dot-like type of calcifications as seen in atherosclerotic disease. As medial calcifications are increasingly considered treatable, our findings may contribute to the development of a treatment strategy for these difficult-to-treat CLI patients.

## Figures and Tables

**Table 1:** Baseline variables of the age-matched CLI patients and the control non-PAD patients.

	Non-PAD (n = 118)	CLI (n = 118)	*p*-Value
Age (years)	71 ± 11	72 ± 12	0.461
Sex (male)	61 (51.7%)	83 (70.3%)	0.003 *
BMI	27.9 (6.0%)	25.2 (3.9%)	0.053
Diabetes mellitus	11 (9.6%)	68 (57.6%)	<0.001 *
History of PAD	0 (0%)	71 (60.7%)	
Stroke	0 (0%)	12 (10.3%)	
CAD	0 (0%)	45 (38.1%)	
Smoking	32 (30.2%)	63 (54.3%)	<0.001 *
eGFR	67 (93)	61 (142)	0.069
Chronic kidney disease (eGFR < 60)	30 (37.5%)	50 (62.5%)	<0.006 *
Severely decreased kidney function (eGFR < 30)	10 (43.5%)	13 (56.5%)	<0.510
Systolic blood pressure	137 ± 20	153 ± 26	<0.001 *
Diastolic blood pressure	78 ± 13	83 ± 12	<0.001 *
Hypertension	54 (45.8%)	64 (69.6%)	<0.001 *
**Any Calcification**			
Crural	66/118 (55.9%)	115/118 (97.5%)	<0.001 *
Femoropopliteal	83/118 (70.3%)	116/118 (98.3%)	<0.001 *

Values are mean ± SD, median (IQR) or n (%) as appropriate. Abbreviations: BMI = body mass index; PAD = peripheral arterial disease; CAD = coronary artery disease; eGFR = estimated glomerular filtration rate (mL/min/1.73 m^2^). * = statistically significant *p*-value.

**Table 2:** Multivariate logistic regression analysis (sex-adjusted) was performed to determine the different calcification characteristics in the crural arteries associated to age-matched non-PAD (n = 65) and CLI patients (n = 115). The patients without calcifications were excluded from this analysis.

Variables in the Equation	OR	95% CI	Standard Error of the Mean	*p*-Value
**Severity**				
Mild	0.55	0.24–1.22	0.391	0.140
Intermediate	0.82	0.37–1.83	0.398	0.627
Severe	1.60	0.87–3.14	0.280	0.122
**Annularity**				
Dot(s)	0.20	0.10–0.41	0.351	<0.005 *
<90°	1.20	0.84–4.69	0.430	0.121
90–270°	0.53	0.15–1.50	0.504	0.231
Complete annularity	2.92	1.55–5.54	0.304	0.001 *
**Thickness**				
Thick (≥1.5 mm)	1.08	0.58–1.98	0.273	0.820
Thin (<1.5 mm)	0.90	0.49–1.66	0.280	0.729
**Continuity**				
Irregular/patchy	1.50	0.79–2.84	0.305	0.212
Continuous	0.86	0.46–1.61	0.293	0.644

* = statistically significant *p*-value.

**Table 3:** Multivariate logistic regression analysis was performed to determine the different calcification characteristics in the femoropopliteal arteries correlated to age-matched non-PAD (n = 83) and CLI patients (n = 116). The patients without calcifications were excluded from this analysis.

Variables in the Equation	OR	95% CI	Standard Error of the Mean	*p*-Value
**Severity**				
Mild	0.44	0.21–0.91	0.366	0.028 *
Intermediate	0.56	0.23–1.36	0.398	0.200
Severe	2.40	1.29–4.48	0.280	0.006 *
**Annularity**				
Dot(s)	0.39	0.18–0.85	0.391	0.019 *
<90°	0.62	0.29–1.32	0.376	0.213
90–270°	1.46	0.79–2.71	0.306	0.232
Complete annularity	1.64	0.90–2.98	0.293	0.105
**Thickness**				
Thick (≥1.5 mm)	1.81	1.09–3.30	0.277	0.053
Thin (<1.5 mm)	0.55	0.30–1.01	0.293	0.053
**Continuity**				
Irregular/patchy	3.27	1.82–5.89	0.283	<0.005 *
Continuous	0.44	0.24–0.81	0.302	0.009 *

* = statistically significant *p*-value.

## Data Availability

The data are not publicly available due to ongoing unpublished research.

## Supplementary Materials

The following are available online at https://www.mdpi.com/article/10.3390/jpm11060493/s1, Table S1: Baseline variables of the sub populations with any calcifications of the initially age-matched CLI patients and the control non-PAD patients, Table S2: Baseline variables of the control group consisted of non-PAD patients, subdivided in patients with and without calcifications in the crural arteries, Table S3: Baseline variables of the control group consisted of non-PAD patients, subdivided in patients with and without calcifications in the femoropopliteal arteries, Table S4: Severity of calcifications in the crural arteries, stratified by age (decades). Table S5: Severity of calcifications in the femoropopliteal arteries, stratified by age (decades).

## Abbreviations

CLI	Chronic limb-threatening ischemia
PAD	Peripheral arterial disease
Non-PAD	Patients without known peripheral arterial disease
OR	Odds ratio
CAD	Coronary artery disease
PADI Trial	Percutaneous transluminal Angioplasty and Drug-eluting stents for Infrapopliteal lesions in critical limb ischemia Trial
PADI Imaging Trial	Percutaneous transluminal Angioplasty and Drug-eluting stents for Infrapopliteal lesions in critical limb ischemia Imaging Trial
RCT	Randomized clinical trial
PTA	Percutaneous transluminal angioplasty
BMS	Bare metal stent
DES	Drug-eluting stent
DM	Diabetes mellitus
eGFR	Electronic glomerular filtration rate (mL/min/1.73 m^2^)
BMI	Body-mass index
