# Supplementary material for: Different Lower Extremity Arterial Calcification Patterns in Patients with Chronic Limb-Threatening Ischemia Compared with Asymptomatic Controls
Source: J Pers Med. 2021 May 31;11(6):493. doi: 10.3390/jpm11060493 (PMC8226835; doi:10.3390/jpm11060493)
Supplement: Supplementary file 1 [file jpm-11-00493-s001.zip › jpm-1193294-supplementary.pdf]

Table S1. Baseline variables of the sub populations with any calcifications of the initially age-matched CLI patients and the control non-PAD patients.

	**Non-PAD**	**CLI**	**p-value**
**Crural**	**66/118 (55.9%)**	**115/118(97.5%)**	**<0.001**
Age (years)	75 (9)	72(11)	
Sex (male)	37(56.9%)	80(69.6%)	0.089
BMI	28(6.07)	25.26(3.94)	0.05*
Diabetes mellitus	9(13.8%)	67(58.3%)	<0.001*
History of PAD	0(0)	69(60.5%)	<0.001*
Stroke	0(0)	12(10.5%)	
CAD	0(0)	45(39.1%)	
Smoking	20(33.9%)	60(54.0%)	0.012*
eGFR	60(76)	61(142)	0.784
Severely decreased kidney function (eGFR<30)	9(40.9%)	13(59.1%)	0.936
Chronic kidney disease (eGFR<60)	26(34.7%)	49(65.3%)	0.117
Hypertension	32(49.2%)	29(82.9%)	0.001*

	**Non-PAD**	**CLI**	
**Femoropopliteal**	**83/118(70.3%)**	**116/118(98.3%)**	**<0.001***
Age (years)	74(8)	72(12)	
Sex (male)	47(56.6%)	82(70.7%)	0.41
BMI	27.9(6.08)	25.24(3.89)	0.05*
Diabetes mellitus	10(12.3%)	67(57.8%)	<0.001*
History of PAD	0(0)	69.0(60.0%)	<0.001*
Stroke	0(0)	12(10.4%)	
CAD	0(0)	45(38.4%)	
Smoking	22(30.6%)	62(54.4%)	<0.001*
eGFR	63(84)	61(142)	
Severely decreased kidney function (eGFR<30)	9(40.9%)	13(59.1%)	0.617
Chronic kidney disease (eGFR<60)	21(30.0%)	49(70.0%)	0.173
Hypertension	38(45.8%)	30(83.3%)	<0.001*

Values are mean ± SD, median (IQR) or n (%) as appropriate.
Abbreviations: BMI=body mass index; PAD=peripheral arterial disease; CAD= coronary artery disease; eGFR=estimated glomerular filtration rate(mL/min/1.73m^2). *= statistically significant p-value.

Table S2. Baseline variables of the control group consisted of non-PAD patients, subdivided in patients with and without calcifications in the crural arteries.

		No calcification				Calcification present			
		**Mean**	**SD**	**N**	**N %**	**Mean**	**SD**	**N**	**N %**
Age		66	11			75	9		
Gender	Male			23	44.2%			38	57.6%
BMI		25.96	4.24			25.59	5.23		
DM				5	9.6%			8	12.1%
PAD				52	100.0%			66	100.0%
Stroke				0	0.0%			0	0.0%
CAD				0	0.0%			0	0.0%
Smoking				11	23.9%			21	35.0%
GFR		73	20			62	22		
Hypertension				21	40.4%			33	50.0%

Table S3. Baseline variables of the control group consisted of non-PAD patients, subdivided in patients with and without calcifications in the femoropopliteal arteries.

		No calcification				Calcification present			
		**Mean**	**SD**	**N**	**N %**	**Mean**	**SD**	**N**	**N %**
Age		66	11			75	9		
Gender	Male			23	44.2%			38	57.6%
BMI		25.96	4.24			25.59	5.23		
DM				5	9.6%			8	12.1%
PAD				52	100.0%			66	100.0%
Stroke				0	0.0%			0	0.0%
CAD				0	0.0%			0	0.0%
Smoking				11	23.9%			21	35.0%
GFR		73	20			62	22		
Hypertension				21	40.4%			33	50.0%

Table S4. Severity of calcifications in the crural arteries, stratified by age (decades).

	Non-symptomatic				Chronic limb-threatening ischemia			
	Severity - crural arteries				Severity - crural arteries			
	**None**	**Mild**	**Intermediate**	**Severe**	**None**	**Mild**	**Intermediate**	**Severe**
Age per decade (years)	Count	Count	Count	Count	Count	Count	Count	Count
40	5	0	1	0	0	2	0	4
50	11	1	0	0	2	3	2	4
60	15	6	3	7	0	7	11	11
70	18	5	2	19	1	3	4	32
80	4	2	5	13	0	0	1	26
90	0	0	1	0	0	0	0	5

Table S5. Severity of calcifications in the femoropopliteal arteries, stratified by age (decades).

	Non-symptomatic				Chronic limb-threatening ischemia			
**Age per decade (years)**	**None (n)**	**Mild (n)**	**Intermediate (n)**	**Severe (n)**	**None (n)**	**Mild (n)**	**Intermediate (n)**	**Severe (n)**
40	6	0	0	0	0	2	0	4
50	8	1	0	3	1	3	2	5
60	8	6	4	13	1	7	4	17
70	11	10	3	20	0	3	4	33
80	2	4	5	13	0	0	0	27
90	0	0	0	1	0	0	0	5
